# Proteome Analysis for Understanding Abiotic Stress (Salinity and Drought) Tolerance in Date Palm (*Phoenix dactylifera* L.)

**DOI:** 10.1155/2015/407165

**Published:** 2015-06-18

**Authors:** Haddad A. El Rabey, Abdulrahman L. Al-Malki, Khalid O. Abulnaja, Wolfgang Rohde

**Affiliations:** ^1^Biochemistry Department, Faculty of Science, King Abdulaziz University, Jeddah, Saudi Arabia; ^2^Bioinformatics Department, Genetic Engineering and Biotechnology Institute, Sadat City University, Sadat City, Minufiya, Egypt; ^3^Max Planck Institute for Plant Breeding Research (MPIPZ), 50829 Cologne, Germany

## Abstract

This study was carried out to study the proteome of date palm under salinity and drought stress conditions to possibly identify proteins involved in stress tolerance. For this purpose, three-month-old seedlings of date palm cultivar “Sagie” were subjected to drought (27.5 g/L polyethylene glycol 6000) and salinity stress conditions (16 g/L NaCl) for one month. DIGE analysis of protein extracts identified 47 differentially expressed proteins in leaves of salt- and drought-treated palm seedlings. Mass spectrometric analysis identified 12 proteins; three out of them were significantly changed under both salt and drought stress, while the other nine were significantly changed only in salt-stressed plants. The levels of ATP synthase alpha and beta subunits, an unknown protein and some of RubisCO fragments were significantly changed under both salt and drought stress conditions. Changes in abundance of superoxide dismutase, chlorophyll A-B binding protein, light-harvesting complex1 protein Lhca1, RubisCO activase, phosphoglycerate kinase, chloroplast light-harvesting chlorophyll a/b-binding protein, phosphoribulokinase, transketolase, RubisCO, and some of RubisCO fragments were significant only for salt stress.

## 1. Introduction

The date palm (*Phoenix dactylifera* L.) is of economic importance to the Kingdom of Saudi Arabia which is the second largest producer of dates worldwide. Although date palms can grow under a variety of environmental conditions, production is impeded by various biotic and abiotic stress factors. Most importantly, water shortage and salinity of the ground water provide abiotic stresses which decrease date production [1]. This is a worldwide problem with some 20% of the world's cultivated land and approximately 50% of all irrigated land being affected. In consequence, adaptation of crop plants to water deficit and salt stress is of high priority in worldwide programmes for breeding modern varieties (for a review see [2]). For date palm thousands of different cultivars are known which have been selected by the producers mainly for improved crop yield and quality [3]. Thus, a high degree of variability is presumably present in date palm germplasms with respect to drought and salinity (desiccation and salt tolerance) [4].

The high salinity exhibits negative effects on the critical biochemical processes of the plant: salt stress affects the whole plant as well as tissues and cells. It can lead to water deficit stress, metabolic toxicity, and nutritional deficiencies and finally drastically reduce production. As studied extensively in* Arabidopsis* and rice [5], three aspects of adaptive responses in plants can be considered under conditions of salt and drought: (a) ion and osmotic homeostasis, (b) growth control/inhibition, and (c) control and repair of stress damage (detoxification). The findings on mechanisms of adaptation to abiotic stresses in model plants such as* Arabidopsis* are relevant to crop plants [2]. Transduction of extracellular, abiotic stress signals via the cell wall and membrane into the cytoplasm and subcellular compartments follows various pathways and triggers various responses. Two of the principal elements in these pathways of plant cells are (i) intracellular Ca^2+^ ions and (ii) protein kinases [6]. Consequently, the sensing of abiotic stress such as drought or salinity results in changes of the phosphorylation status of cellular proteins [7]. As revealed by mutant analysis in* Arabidopsis*, abscisic acid (ABA) is another key regulator of signal cascades that are triggered by salt and water deficit [8].

Molecular studies in date palm for understanding some basic molecular mechanisms in response to drought and salinity have been rather limited [9–17]. Recent date proteome analyses, however, open the way to the identification of important biomarkers. Dakhlaoui-Dkhil et al. [18] identified an ABC superfamily ATP-binding cassette transporter as a putative biomarker for male date palms. Palms affected by the leaf brittle disease express a manganese-stabilizing 33 kDa protein not detectable in healthy plants [19]. The recent sequencing and annotation of date palm genomes [20] opens the possibility for the application of further high throughput technologies to the study of stress-related gene functions in date palm. Proteome analysis, for example, opens the possibility to identify date palm proteins involved in transduction network regulation via posttranslational protein modification by phosphorylation/dephosphorylation under abiotic stress conditions [21] as studied for example, in the desiccation-tolerant plant* Craterostigma plantagineum* [22, 23].

The effort described here relates to proteome analysis of salinity and water stress-related sensitive protein resulting from the salinity and drought gene expression in leaves of young date palm seedlings.

## 2. Materials and Methods

### 2.1. Date Palm Cultivation

Seeds of the cultivar “Sagie” were scarified with sulfuric acid (96%) for 5 min and washed 5 times with sterile distilled water, followed by sterilization with 1% (v/v) mercuric chloride for 3 min, washed 5 times with sterile distilled water, and imbibed for 48 h in distilled water. The seeds were sterilized a second time with calcium hypochlorite (5%, v/v) for 4 min and washed 4 times with sterile distilled water. Seeds were germinated between wet layers of tissue papers until the radical reached 1 cm and then transferred to pots containing organic soil and irrigated with tap water and grown in growth chambers under controlled light conditions (12 h light/12 h dark) at 30°C until the age of three months.

### 2.2. Stress Experiments

Twelve 3-month-old date palm seedlings were selected and divided as follows: 4 seedlings were daily irrigated with distilled water for one month as control, 4 seedlings were subjected to drought (27.5 g/L PEG 6000) for one month, and the other 4 seedlings were subjected to salt stress with 16 g/L NaCl, according to a modified method of Sané et al. [24].

At the end of the stress period, samples were washed with distilled water, frozen in liquid nitrogen, and stored at −80°C until use.

### 2.3. Protein Extraction

Four replicates of the frozen shoot materials of stressed and control plantlets were ground into a fine powder under liquid nitrogen, and the proteins were precipitated by the addition of 1.8 mL of ice-cold acetone containing 0.07% (v/v) mercaptoethanol. The raw precipitates were dried by lyophilisation and stored at −80°C for further processing.

One hundred mg of each of the lyophilized raw extract was dissolved in 400–600 *μ*L of IEF buffer (7 M urea, 2 M thiourea, 2% (w/v) CHAPS, and 30 mM Tris pH 8.0). The proteins were resolubilized overnight at room temperature (RT). The mixture was then centrifuged for 10 minutes at 4°C at 16,100 ×g and total soluble protein in the supernatants was quantified using the 2D Quant Kit (GE Healthcare, Munich, Germany). Equal amounts of all single samples were pooled to get DIGE internal standard (IS). Amount of 50 *μ*g of IS was used for each analytical gel and 300 *μ*g of IS for each preparative gel (necessary for protein identification by MS).

### 2.4. Protein Labeling and 2D Electrophoresis

50 *μ*g of each protein sample as well as needed amount of internal standard was labeled with fluorescent dyes using the Refraction-2DTM Labeling Kit (NH DyeAGNOSTICS GmbH, Halle, Germany) according to the manufacturer's protocol. The internal standard was labeled with G100, whereas the single analyzed samples were labeled with G200 or G300 before mixing.

2D gel electrophoresis was briefly performed as follows: samples mixture was separated in the first dimension according to their isoelectric point (pI) using immobilized pH gradient strips (Immobiline DryStrip, 24 cm, pH 4–7, GE Healthcare) focused by IPGphor 3 (GE Healthcare) and in the second dimension according to their molecular weight by SDS-PAGE using the Ettan DALT*twelve* gel system (GE Healthcare). For preparative gels, glass plates were silanized on one side prior to gel casting and the gels were run in parallel to the analytical gels. The fluorescent scans of the analytical gels were generated using Ettan DIGE Imager (GE Healthcare) immediately after electrophoresis. Preparative gels were stained with 1 mM ruthenium(II)-tris(bathophenanthroline disulfonate) fluorescence stain and reference markers were attached to the glass plates prior to scanning, thus enabling blind picking of the protein spots after the difference gel electrophoresis (DIGE) analysis. The preparative gels were scanned directly after destaining and stored wet at 4°C before spot cutting.

### 2.5. DIGE Analysis

The gel images were processed with DeCyder Software v7.0 (GE Healthcare). The internal standard included all proteins in the analysis and as it was run on every gel along with all analyzed samples, it was used for spot matching across all the gels. The biological variation analysis (BVA) module allowed quantitative comparisons of protein expression across multiple gels. The extended data analysis (EDA) was used for multivariate analysis of protein expression data derived from the BVA module in order to perform a principal component analysis (PCA) and to identify protein spots of interest with differential expression analysis. All automatically identified spots were checked manually to confirm that they are real spots and marked for picking.

### 2.6. Mass Spectrometry (MS) Analysis

For protein identification in a single spot, proteins fixed in the polyacrylamide gel plug were reduced, alkylated, and digested with trypsin (Promega, Mannheim, Germany). The resulting peptides were analyzed by nano-HPLC (UltiMate 3000 HPLC System, LC Packings, Dionex, Idstein, Germany) coupled to an amaZon ETD MS ion trap spectrometer (Bruker Daltonics, Bremen, Germany) using nano-ESI spray. The nano-HPLC system and the ion trap spectrometer were controlled using the Bruker Compass HyStar v3.2-SR2 software. The liquid chromatography system was supplied with reversed-phase precolumn (LC Packings, Dionex) for sample desalting and a 15 cm PepMap 100 reversed-phase C18 column, 75 *μ*m inner diameter (LC Packings, Dionex), for peptide fractionation. The peptides were separated using a 45 min linear gradient from 96% (v/v) solution A (2% (v/v) acetonitrile, 0.1% (v/v) formic acid in high purity water) and 4% (v/v) solution B (98% (v/v) acetonitrile, 0.1% (v/v) formic acid in high purity water) to 50% (v/v) solution A and 50% (v/v) solution B at a flow rate of 300 nL/min. The electrospray was operated in positive ion mode with −4000 V spray voltage and 10 psi gas pressure. The end plate offset of the mass spectrometer was set to −500 V and for the acquisition the standard method Proteomics Auto MSMS Alternating Spectra CID-ETD Bruker trap Control v7.0 was used. Raw data files were evaluated using Compass Data Analysis v4.0-SR5 Software with embedded search engine Mascot Search 2.3.01 (Matrix Science Ltd., London, UK). Swiss Prot (All species) and NCBInr (Green plants) databases were involved in the protein search using the following parameters: enzyme trypsin, up to one missed cleavage permitted, no fixed modifications and variable modifications carbamidomethyl (C), oxidation (M) and propionamide (C) allowed, and mass tolerance for both precursor ion and fragment ion ±0.3 Da. Only the protein hit with highest protein score was used for further analysis. When the protein was identified with one peptide only or several proteins with similar protein score were identified in a spot, the spots were excluded from the analysis.

## 3. Results

### 3.1. The Difference Gel Electrophoresis (DIGE)

The objective of this experiment is comparison of protein spots of salt-stressed and drought-stressed date palm shoots by searching for new protein spots or proteins spots differing in their intensity due to stress. In this experiment, 4 salt stress samples (61–64), 4 drought stress samples (73–76), and 4 control samples (1–4) of lyophilized raw protein extracts of date palm were analyzed. Samples 61–64 represent the date palm seedlings that were exposed to a high concentration of NaCl (16 g/L) for one month, whereas samples 73–76 represent seedlings that were exposed to a high PEG concentration (27.5 g/L) for the same period of time. All the samples were solubilized in IEF1 buffer (7 M urea, 2 M thiourea, 2% CHAPS, and 30 mM Tris pH 8.0) according to the data shown in Table 1. Different buffer volumes were used for each sample to be able to be resolubilized. After overnight solubilization and subsequent centrifugation, the soluble proteins were quantified using the 2D Quant kit. Estimated total protein concentrations are listed in Table 1.

For successful labelling of the samples with G200 or G300, all the samples were diluted with IEF 1 buffer to reach the concentration of the sample with lowest concentration (1.1 *μ*g/*μ*L in this case, Table 1). An internal standard was prepared by mixing all 12 samples in the same weight ratio and the standard was labelled with G100.

The presence of an internal standard in every gel provided an intrinsic link between samples. Each protein spot in a sample was compared to its representative spot within the internal standard on the same gel to generate a ratio of relative protein levels. Quantitative comparison of samples between gels was based on the relative change of a sample to its in-gel internal standard. The labelling and mixing scheme for the performed DIGE experiments is shown in Table 2. Six analytical gels were run in the pH range of 4–7. Part of the internal standard was saved before labelling with G100 and this unlabeled part was applied to 2 preparative gels that were in the end used for the protein identification by mass spectrometry. Proteins from the extracts were separated in the first dimension according to their protein intensity using IEF on Immobiline DryStrip, 24 cm, pH 4–7, and in the second dimension according to their molecular weight using SDS-PAGE. All of the 6 analytical gels and the 2 preparative gels were run at the same time.

After the run, the gels were scanned. Three fluorescence scans for each analytical gel were acquired and used for the analysis. The preparative gels were fixed for 1 hour in fixation solution (40% ethanol and 10% acetic acid in water) and then stained with RuBPS (1 mM in fixation solution) for 20 minutes and destained overnight in fixation solution. The preparative gels were scanned directly after destaining and stored wet at 4°C before spot cutting.

### 3.2. Results of 2D-Gel Electrophoresis

(See Figure 1).

### 3.3. DIGE Analysis

#### 3.3.1. Principal Component Analysis (PCA)

**Figure 2:**
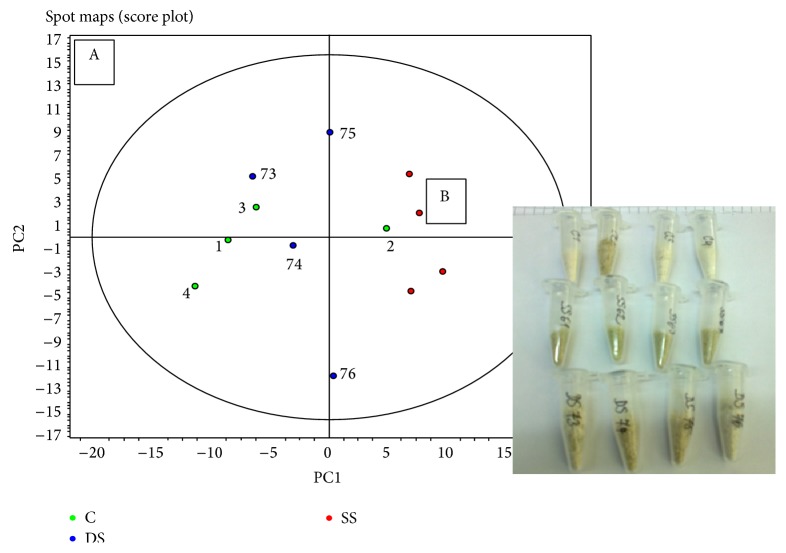
(A) Principle component analysis (PCA): red circles show the position of salt stress spot maps and blue drought stress spot maps and green circles are the controls; numbers indicate the corresponding sample numbers. (B) Photo of raw protein extracts before solubilization: in the upper row are the controls, in the middle one salt stress samples, and in the lower row the drought stress samples, all of them in chronological order from left to right.

For DIGE analysis, spots were detected on all scans and intergel matching was performed using the DeCyder software. The matching was manually checked and corrected in mismatched regions. In the next step, a PCA analysis was performed. PCA helps to identify some underlying sources of variation and gives first impression on how well experimental groups can be separated. Spots that could be localized on 80% of spot maps (gel scans) were included in the analysis (Figure 2).

*The Quality Check of the Biological Replicates.* Scores of the spot maps for salt stress (red circles in Figure 2(A)) were localized closely to each other in the graph and thus showed a good reproducibility for the salt stress biological replicates. In addition, scores of the spot maps for controls (green circles in Figure 2(A)) had an outlier in spot map for sample 2. Other three spot maps were well separated from the salt stress spot maps in a compact group. Furthermore, scores of the spot maps for drought stress (blue circles in Figure 2(A)) were located between controls and salt stress with an outlier for sample 76. Position of other three replicates to each other indicated a lower reproducibility of the drought stress compared to the salt stress. Position closer to the control suggested smaller differences in drought stress as compared to salt stress.

#### 3.3.2. Differential In-Gel Analysis (DIA): Looking for the Differences in Spot Pattern Comparing Fluorescence Scans Coming from the Same Gel

**Figure 3:**
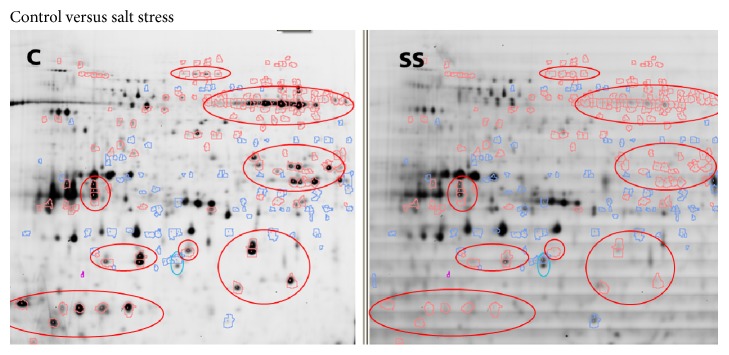
Fluorescence scans of control (C) versus salt stress (SS): spots shown in red had higher standardized abundance in control compared to salt stress and blue color highlighted the spots with lower abundance. Red marked regions contained intensive spots significantly decreased in abundance in salt stress sample, and the blue one shows increased abundance.

**Figure 4:**
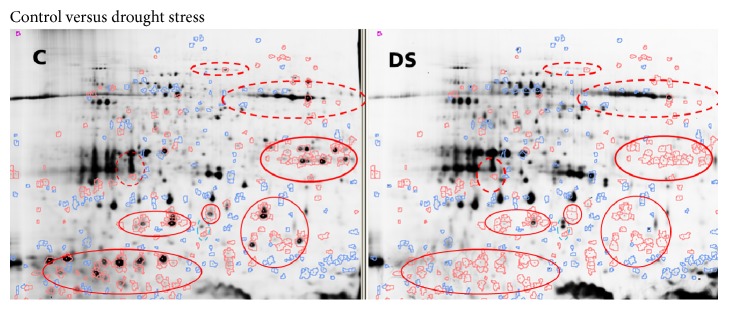
Fluorescence scans of control (C) versus drought stress (DS): spots shown in red had higher standardized abundance in control compared to drought stress and blue color highlighted the spots with lower abundance. Red marked regions contained intensive spots significantly decreased in abundance in drought stress sample. Dashed lines indicate the regions important in salt stress but not changing significantly in drought stress.

**Figure 5:**
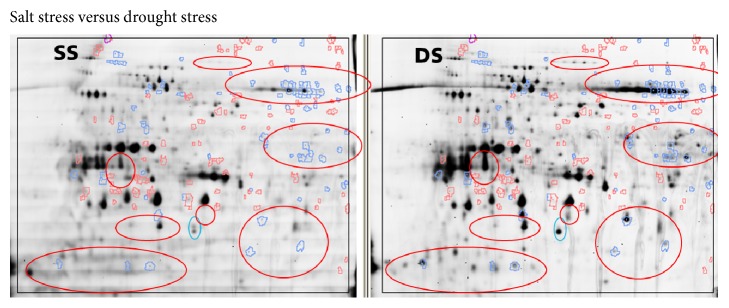
Fluorescence scans of salt stress (SS) versus drought stress (DS): spots shown in red had higher standardized abundance in salt stress compared to drought stress and blue color highlighted the spots with lower abundance. Red and blue marked regions contained intensive spots significantly decreased and increased in abundance in salt stress sample. The red regions with blue highlighted spots indicated more significant changes (decrease) for salt stress and regions without any highlighted spots indicated comparable changes for both stresses.

For this analysis, the standardized abundance of each spot on the gel was calculated for two samples in the gel using the spot intensities of the internal standard. Significant differences between the samples were visualized showing blue or red spot contours for higher or lower standardized abundance (threshold 3) of the spots, respectively. Regions with intensive spots changing significantly were manually marked.  Figure 3 shows fluorescence scan of the control versus salt stress, Figure 4 shows fluorescence scan of control versus drought stress, and Figure 5 shows fluorescence scan of the salt stress versus drought stress.

Several regions with significant spot changes were localized and may indicate potential effects on the proteome due to stress. Most of the changed spots were decreased in the relative abundance due to stress. Only few mostly less abundant spots showed increased intensity after stress.

#### 3.3.3. Biological Variation Analysis (BVA) and Extended Data Analysis (EDA)

**Figure 6:**
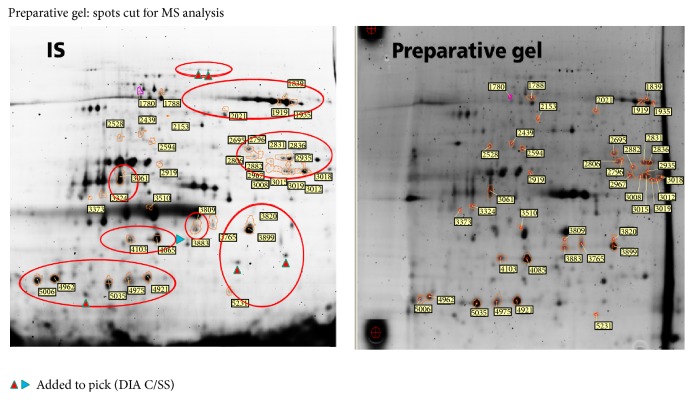
Spots chosen for picking: IS-internal standard scan shows all picked spots marked with red spot contours and yellow number boxes and spots marked with colored arrows were added to the pick from DIA. Red marked regions correspond to the regions found in DIA for salt stress (Figure 5). On the scan of the preparative gel, the same spots are highlighted. The figure illustrates good match of analytical and preparative gel.

BVA allowed quantitative comparisons of protein expression across multiple gels. *t*-test (*P* value calculated using Student's *t*-test), one-way ANOVA (*P* value calculated using one-way ANOVA statistical test), and average ratio (average ratio between the groups selected in the protein statistics) values were calculated for all matched spots. The spots were filtered for one-way ANOVA value lower than 0.2, *t*-test value lower than 0.1, and average ratio <2 and >−2. EDA is proteomic software for multivariate analysis of protein expression data derived from BVA module. It was used for PCA analysis presented in Section 1 and to find the protein spots of interest with differential expression analysis. All automatically chosen spots were checked manually if they are real spots and marked for picking (Figure 6).

Using low level statistical parameters, 47 spots were chosen for protein identification, most of them with lower spot volume under stress conditions compared to the standard. Detailed results of DIGE analysis are found in the supplementary excel tables (in Supplementary Material available online at http://dx.doi.org/10.1155/2015/407165).

### 3.4. Protein Identification of Stress Sensitive Proteins in Using Mass Spectrometry (MS)

#### 3.4.1. Identification of Proteins in the Spots of Interest Chosen by DIGE Analysis

**Figure 7:**
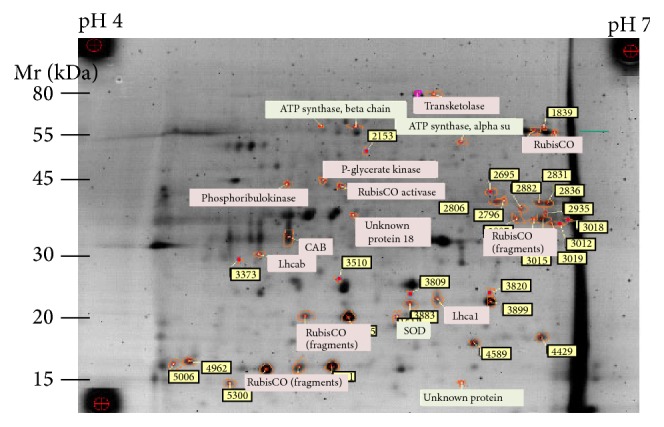
The identified proteins shown on the scan of preparative gel. Proteins in the spots marked with the red circle could not be identified. The color code used in protein name boxes is the same as in Table 3.

Forty-seven spots were cut from the preparative gel for MS analysis. Proteins in the gel plugs were reduced with 10 mM dithiothreitol and alkylated using 55 mM iodoacetamide in 0.1 M to open S-S bridges for action of trypsin. Digestion with trypsin (12.5 ng/*μ*L of trypsin in 50 mM NH_4_HCO_3_) was performed overnight at 37°C. The resulting peptides were extracted from the gel plugs in two extraction steps: first one with 25 mM NH_4_HCO_3_ and second one with 5% formic acid. Collected extracts were dried down and resolubilized in 2% acetonitrile with 0.1% formic acid in water (MS grade) for MS analysis. The resulting peptides were separated according to their hydrophobicity by nano-HPLC (C18 column, UltiMate* 3000 HPLC* system, Dionex) and sprayed directly into an ion trap spectrometer (amaZon ETD, Bruker Daltonics) using nano-ESI sprayer. Processed MS/MS spectra were used for the protein identification with in-house Mascot Search server (Matrix Science software). Swiss Prot (All species) and NCBInr (Green plants) databases were involved in the protein search (Figure 7 and Table 3).

*MS Results.* Biological replicates for salt stress showed good reproducibility and were well separated from control samples in the PCA score plot. Drought stress replicates showed lower reproducibility and differed less from the control. Several regions with significant spots changing probably due to the applied stress were localized in the gel scans using differential in-gel analysis (DIA, DeCyder). Most of the spots from these regions were decreased in the relative abundance under stress conditions. Only low quality statistics allowed the finding of some more intensive spots sensitive to stress. Proteins in 47 spots were analyzed by mass spectrometry; for 8 spots no protein could be identified. Levels of ATP synthase alpha and beta subunits, unknown protein 18 and some of RubisCO fragments were significantly changed under both stress conditions. Changes in abundance of superoxide dismutase, chlorophyll A-B binding protein, light-harvesting complex I protein Lhca1, RubisCO activase, phosphoglycerate kinase, chloroplast light-harvesting chlorophyll a/b-binding protein, phosphoribulokinase, transketolase, RubisCO, and some of RubisCO fragments were significant only for salt stress.

## 4. Discussion

Proteome analysis provides one of the best options for the functional analysis of translated regions of the genome. The levels of ATP synthase alpha (Accession number gi|158325128) and beta subunits (Accession number gi|292559515), unknown protein (Accession number gi|205830697), and some of RubisCO fragments (Accession numbers gi|28195663, gi|292559516, gi|3152721, gi|209417491, gi|209417489, gi|55785631, and gi|16565309) were significantly changed under both stress conditions compared to the control which indicates that these protein subunits are associated with drought and salinity stress [25–27].

In the current study, changes in abundance of superoxide dismutase, chlorophyll A-B binding protein, light-harvesting complex I protein Lhca1, RubisCO activase, phosphoglycerate kinase, chloroplast light-harvesting chlorophyll a/b-binding protein, phosphoribulokinase, transketolase, RubisCO, and some of RubisCO fragments were significant only in salt stress condition. This result is consistent with many salinity stress tolerance studies in other organisms [28–30].

The superfamily of light-harvesting chlorophyll a/b-binding (Lhc) proteins in higher plants and green algae is composed of more than 20 different members associated with photosystem I (PSI) or photosystem II (PSII) [31]. In this study, chlorophyll A-B binding protein (CAB) and light-harvesting complex I protein Lhca1 were upregulated. Accumulation of CAB and Lhca1 in PSI exposed to salt and drought stress might represent one of the strategies to prevent or lower light stress-induced damage [25, 26, 28]. The accumulation of Chl has been proposed as one of the potential biochemical indicators of salt tolerance in different crops [30].

It was also proposed that these proteins might have a protective function within PSII under stress conditions either by binding free chlorophyll molecules and preventing the formation of free radicals and/or by acting as sinks for excitation energy, because under stress conditions, a mobile pool of Lhcb1 and Lhcb2 moves from PSII to PSI due to the reversible phosphorylation of Lhc proteins by a thylakoid-bound kinase [26, 32]. Thylakoid membrane proteins were also affected by salt stress [33]. Decrease in the initial activity and activation state of RubisCO as a result of drought stress was encountered in Mediterranean species [29].

In the present study, the expression of superoxide dismutases (SOD) was changed under salt and drought condition. The superoxide dismutases are metalloenzymes that catalyze the dismutation of ion superoxide into oxygen and hydrogen peroxide (SODs) and constitute the first line of defence against reactive oxygen species [34]. The superoxide radical is a reactive oxygen species (ROS) whose production increases under abiotic and biotic stresses, including drought [28, 35]. Meanwhile, Alscher et al. [34] stated that SODs play a critical role in protecting plant tissues from ROS. Roveda-Hoyos and Fonseca-Moreno [28] reported that eight proteins were overregulated in wheat leaves. In response to salt stress, among them a protein complex of PSII, a protein OEE2 (oxygen-evolving enhancer protein 2), and superoxide dismutase. They added that the latter enzyme has also been reported in response to drought.

The change in regulation of phosphoribulokinase in the current study is also correlated with salt stress. The expression of PRK (phosphoribulokinase) in ice plant was affected under salt stress conditions. The amount of mRNA was declined by a factor of approximately three within days, followed by an increase to approximately prestress levels [36].

In conclusion, proteome of salinity and drought-stressed palm seedlings was compared with that of nonstressed plants of the same age. DIGE analysis identified 47 sensitive proteins associated with the stress condition. The MS analysis of 47 sensitive protein spots showed that 12 proteins could be identified, 3 of them were significantly changed in both stresses, and 9 proteins were significantly changed only in salt-stressed plants. Proteins could not be identified in 8 spots, whereas in 26 spots RubisCO and its fragments were identified.

## Supplementary Material

Supplementary materials shown in Table (1) is DIGE and MS analysis, Table (2) shows average ra-tio of robustly identified proteins, Table (3) shows robustly identified proteins (in salt stress, drought stress and salt and drought stress), Table (4) shows t-test for robustly identified proteins and Table (5) Average ratio of robustly identified proteins in drought and salt stressed plants.

## Figures and Tables

**Figure 1:**
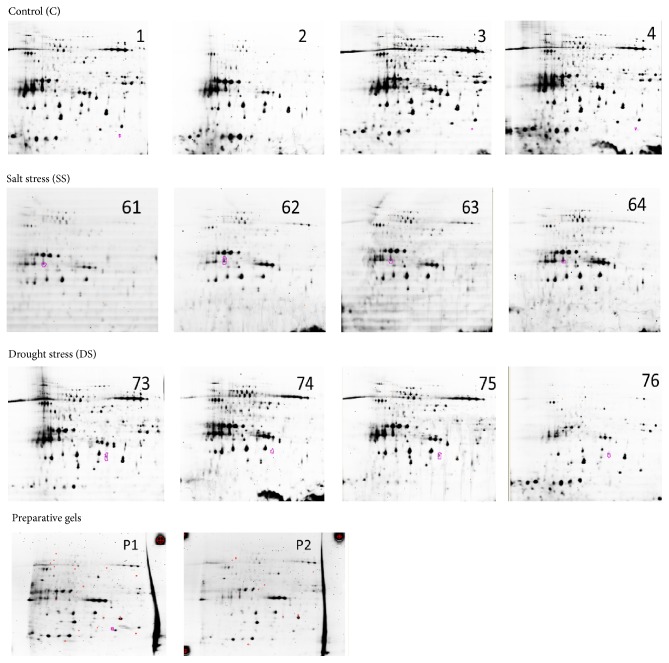
Overview of the fluorescence scans: the scans are labeled with corresponding sample numbers and P1 and P2 are preparative gel replicates.

**Table 1:** Amount of the dry samples (*m*), volume of the IEF1 buffer added for protein solubilization (IEF1), and the reached protein concentration of the resolubilized samples (*c*) are listed (bold are control samples, italic are salt stress samples, and bold italic are drought stress samples).

Treatment	Sample number	*m* [mg]	IEF1 [*μ*L]	*c* [*μ*g/*μ*L]
Control	**1**	**100**	**600**	**5.0**
Control	**2**	**100**	**500**	**3.6**
Control	**3**	**100**	**600**	**4.5**
Control	**4**	**100**	**600**	**3.3**
16 g/L NaCl	*61 *	*100 *	*400 *	*1.5 *
16 g/L NaCl	*62 *	*100 *	*400 *	*1.1 *
16 g/L NaCl	*63 *	*100 *	*400 *	*1.5 *
16 g/L NaCl	*64 *	*100 *	*400 *	*1.1 *
27.5 g/L PEG	***73***	***100***	***600***	***5.1***
27.5 g/L PEG	***74***	***100***	***400***	***3.6***
27.5 g/L PEG	***75***	***100***	***500***	***4.5***
27.5 g/L PEG	***76***	***100***	***600***	***3.3***

**Table 2:** Labeling scheme for 6 gels: The internal standard (IS) as well as each analyzed sample was labeled with G-dyes G100, G200, or G300 as shown in the table (bold are control samples, italic are salt stress samples, and bold italic are drought stress samples). The internal standard is the mixture of the same portions (*w*) of all analyzed samples.

Gel number	G100	G200	G300
1	IS	**1**	*61 *
2	IS	**2**	***73***
3	IS	*62 *	**3**
4	IS	***74***	**4**
5	IS	***75***	*63 *
6	IS	*64 *	***76***

**Table 3:** Identified proteins (when the protein was found in several spots only the most intensively changed spot is listed; the exception is RubisCO fragments). (A) Spots with higher abundance in stress compared to control. (B) Spots with lower abundance. The spots written in bold were significantly changed in both stresses. The rest of the spots changed significantly only in salt stress.

Spot number	Average ratio SS	Average ratio DS	Protein name (organism)	Molecular weight (kDa)	Protein score	Number of peptides
(A) Spots with higher abundance in stress compared to control						
**2021**	**3.32**	**2.02**	**ATP synthase alpha subunit (*Phoenix dactylifera*)**	**18618**	**99**	**1**
**1788**	**3.3**	**2.03**	**ATP synthase CF1 beta chain (*Phoenix dactylifera*)**	**53847**	**1339**	**18**
4041	2.45	1.12	Superoxide dismutase [Cu-Zn], chloroplastic OS = Vitis vinifera GN = SODCP PE = 2 SV = 1	21.7	117	2

(B) Spots with lower abundance in stress compared to control						
3061	−2.6	−1.82	Chlorophyll A-B binding protein (CAB), putative (*Musa acuminata*)	28055	152	2
3765	−2.71	−1.73	Light-harvesting complex I protein Lhca1 (*Populus trichocarpa*)	26532	136	2
**2919**	−2.96	−2.03	**Unknown protein 18**	**1393**	**125**	**2**
**2796**	−4.12	−2.5	**Ribulose-1,5-bisphosphate carboxylase/oxygenase large Subunit-fragment (*Phoenix dactylifera*)**	**49566**	**369**	**2**
**3008**	−2.57	−3.7	**Ribulose-bisphosphate carboxylase-fragment (*Cyrtosperma macrotum*)**	**51852**	**229**	**2**
2594	−3.99	1.33	Ribulose bisphosphate carboxylase/oxygenase activase, Chloroplastic-like (*Setaria italica*)	52132	235	3
2439	−4.39	−1.62	Phosphoglycerate kinase, putative (*Ricinus communis*)	50114	431	5
3324	−4.57	−1.6	Chloroplast light-harvesting chlorophyll a/b-binding Protein (*Artemisia annua*)	28542	90	2
2528	−4.67	−1.27	Phosphoribulokinase (*Zea mays*)	46078	176	2
1278	−5.15	−1.63	Transketolase (*Camellia sinensis*)	81266	268	2
1935	−7.32	−1.53	Ribulose-1,5-bisphosphate carboxylase/oxygenase large Subunit (*Harpagophytum grandidieri*)	52139	153	2
2836	−9.99	−1.74	Ribulose-1,5-bisphosphate carboxylase/oxygenase large Subunit-fragment (*Phoenix dactylifera*)	49566	628	6
4921	−13.07	−1.88	Ribulose-1,5-bisphosphate carboxylase/oxygenase large Subunit-fragment (*Burchellia bubalina*)	52367	252	3
